# Buffalo Medical and Surgical Association

**Published:** 1885-10

**Authors:** 


### The Buffalo Medical and Surgical Association.

For many years, indeed since its organization in 1856, the *Journal* has published monthly the proceedings of the Association. Both of its present editors are ex-presidents of the Society, and nearly all of its former editors have at some time filled the presidential chair. The *Journal* has been ever mindful of the interests of the Association, and we have labored with diligence for its prosperity and success, believing that it was an important factor in the promotion of honorable fellowship, the advancement of medical science, and the maintenance of a high professional standard among the physicians of Buffalo. During the past thirty years, the Society has passed through several periods of great prosperity, and again has had a mere languishing existence. Three years ago, when it cut loose from even the appearance of a connection with a medical college, the Association took on a new life, its meetings have been largely attended, harmony and good fellowship prevailed, and some creditable work accomplished. A few weeks ago, we looked forward with the reasonable hope that, under its present able President, the current year would be the most fruitful of good work in the history of the Society.

In marked and painful contrast with such anticipation, was the condition of things manifested at the last meeting of the Association. Out of a membership of 97, there were but six members present with the President and Secretary.

The essayist did not feel called upon to read his paper to such an audience, and the meeting ended with the trifling results indicated in the minutes which appear in this number.

The profession in the city do not need to be informed as to the cause of this decline. Our non-resident readers would not be interested in the details of a three hours' angry debate on a subject introduced for the gratification of personal enmity and partizan spite; the charge being made that the medical staff of one of the great hospitals of the city had in their annual report concealed deaths. A motion made to lay the whole matter upon the table was lost by a tie vote, 27 to 27, and the ridiculous affair ended by the carrying of a motion to the effect that all hospitals should exercise due care in the tabulation of their statistics.

We wish greatly that we could say that the whole matter did entirely end there; but "behold how great a matter a little fire kindleth." In this case, it was a little fire truly. The charge, or rather insinuation, made by a bellicose individual, whose chief pleasure, it would appear to be, is to make discord, was totally unsustained, and had a moment's weight only because, with an indignation which under the circumstances was natural, the gentlemen accused refused to pay any attention to the charge or to recognize the right of the Society to question their honesty on a mere insinuation. This refusal was made much of by certain members of the staff of a rival hospital in communications to the secular press, until the hospital interested published the names of every individual who had died in the hospital during the time included in the report, and most conclusively proved the falsity of the charge. The discussion of the case at the subsequent meeting of the Association evolved so much bitterness and angry feeling, and that among the oldest and most respected members, that probably a majority of those present left the rooms of the Society, vowing that they would never attend another meeting.

We must earnestly hope, however, that wiser counsels will prevail. Those who felt themselves aggrieved are among those who have supported the Association most heartily—and some of them for a life-time. Without them, the Association wou d no longer represent the profession in Buffalo, but simply one of the medical colleges. If another association is formed, it will probably largely represent the other medical college. There is no objection to such organizations being formed; but there is a need for the existence in the city of an association fairly representing the profession, and not any mere party or clique; and the true functions of such a society are to afford a meeting-place of the various sections of the profession upon neutral ground, and thus to diffuse knowledge and to present a worthy field for the presentation of original work; to survey, from time to time, by the light of mutual reflection, the real progress of medical science, and thus to seek sound guidance in the application of our knowledge to our practical duties.

It is only by united action that these desirable ends can be reached, and we believe that, as a result of the unseemly wrangle at the August meeting, the introduction into the Association of subjects foreign to medical science, and solely in the interests of rival hospitals or colleges, will never again be allowed. With this belief, and an eye single to the best interests of the profession, we would ask all the members of the Association to overlook personal grievances, and hereafter, as in the past, labor to advance the true interests of the profession and the Association.

